# Targeting the epidermal growth factor receptor in non-small cell lung cancer cells: the effect of combining RNA interference with tyrosine kinase inhibitors or cetuximab

**DOI:** 10.1186/1741-7015-10-28

**Published:** 2012-03-21

**Authors:** Gang Chen, Peter Kronenberger, Erik Teugels, Ijeoma Adaku Umelo, Jacques De Grève

**Affiliations:** 1 Laboratory of Medical and Molecular Oncology and Department of Medical Oncology, Universitair Ziekenhuis Brussel, Vrije Universiteit Brussel, Laarbeeklaan 101, 1090, Brussels, Belgium; 2 Department of Pathology, First Affiliated Hospital, Guangxi Medical University, Shuangyong Road 6, 530021, Nanning Guangxi, People's Republic of China; 3 Laboratory for Biotechnology, Department of Gezondheidszorg, Erasmushogeschool Brussel, Laarbeeklaan 125, 1090, Brussels, Belgium

**Keywords:** EGFR, RNA interference, tyrosine kinase inhibitors (TKIs), anti-EGFR monoclonal antibodies (mAbs), proliferation, apoptosis, lung cancer

## Abstract

**Background:**

The epidermal growth factor receptor (EGFR) is a validated therapeutic target in non-small cell lung cancer (NSCLC). However, current single agent receptor targeting does not achieve a maximal therapeutic effect, and some mutations confer resistance to current available agents. In the current study we have examined, in different NSCLC cell lines, the combined effect of RNA interference targeting the EGFR mRNA, and inactivation of EGFR signaling using different receptor tyrosine kinase inhibitors (TKIs) or a monoclonal antibody cetuximab.

**Methods:**

NSCLC cells (cell lines HCC827, H292, H358, H1650, and H1975) were transfected with EGFR siRNA and/or treated with the TKIs gefitinib, erlotinib, and afatinib, and/or with the monoclonal antibody cetuximab. The reduction of EGFR mRNA expression was measured by real-time quantitative RT-PCR. The down-regulation of EGFR protein expression was measured by western blot, and the proliferation, viability, caspase3/7 activity, and apoptotic morphology were monitored by spectrophotometry, fluorimetry, and fluorescence microscopy. The combined effect of EGFR siRNA and different drugs was evaluated using a combination index.

**Results:**

EGFR-specific siRNA strongly inhibited EGFR protein expression almost equally in all cell lines and inhibited cell growth and induced cell apoptosis in all NSCLC cell lines studied, albeit with a different magnitude. The effects on growth obtained with siRNA was strikingly different from the effects obtained with TKIs. The effects of siRNA probably correlate with the overall oncogenic significance of the receptor, which is only partly inhibited by the TKIs. The cells which showed weak response to TKIs, such as the H1975 cell line containing the T790M resistance mutation, were found to be responsive to siRNA knockdown of EGFR, as were cell lines with downstream TKI resistance mutations. The cell line HCC827, harboring an exon 19 deletion mutation, was more than 10-fold more sensitive to TKI proliferation inhibition and apoptosis induction than any of the other cell lines. Cetuximab alone had no relevant *in vitro *activity at concentrations obtainable in the clinic. The addition of EGFR siRNA to either TKIs or cetuximab additively enhanced growth inhibition and induction of apoptosis in all five cell lines, independent of the EGFR mutation status (wild-type or sensitizing mutation or resistant mutation). The strongest biological effect was observed when afatinib was combined with an EGFR-specific siRNA.

**Conclusions:**

EGFR knockdown by siRNA further decreases the cell growth of lung cancer cells that are treated with TKIs or cetuximab alone, confirming that single agent drug targeting does not achieve a maximal biological effect. The siRNA inhibits EGFR oncogenic activity that bypasses downstream "resistance" mutations such as KRAS and PTEN. The combined treatment of siRNA and EGFR inhibitory agents is additive. The combination of a potent, irreversible kinase inhibitor such as afatinib, with EGFR-specific siRNAs should be further investigated as a new strategy in the treatment of lung cancer and other EGFR dependent cancers, including those with downstream resistance mutations.

## Background

Non-small cell lung cancer (NSCLC) comprises 75% to 85% of newly diagnosed lung cancers. Over 70% of NSCLC patients present with advanced disease, and the 5-year survival rate for NSCLC is only 16%. For early-stage or locally-advanced lung cancer, surgery is the most effective treatment, and combined chemotherapy is the standard adjuvant approach. For stage III/IV NSCLC, platinum-based combined chemotherapy is the current standard of care, but with much room for improvement [1].

In a minority of patients, a mutant epidermal growth factor receptor (EGFR) has become a validated therapeutic target and EGFR tyrosine kinase inhibitors (TKIs) gefitinib and erlotinib are currently the first-line treatment options for these patients [2,3]. These drugs lead to impressive improvements in progression-free survival (PFS) compared to chemotherapy. However, ultimately these tumors develop resistance to these TKIs through various mechanisms. A frequent mechanism is the emergence of a malignant clone with a second mutation in the EGFR kinase domain, a threonine-to-methionine substitution at amino acid position 790 (T790M) [4].

The ErbB family includes four related receptor proteins (EGFR/ErbB1/HER1, ErbB2/Neu/HER2, ErbB3/HER3, and ErbB4/HER4). The ErbB family of membrane receptors is a group of transmembrane glycoproteins that consists of an extracellular ligand-binding domain, a transmembrane domain, and an intracellular tyrosine kinase domain mediating signal transduction. The complex EGFR signal transduction pathway involves the RAS/MAPK cascade, phosphatidyl inositol 3-kinase (PI3K), signal transducer and activator of transcription (STAT), and downstream protein kinase C (PKC). Following ligand binding, EGFR can homodimerize or heterodimerize with another member of the ErbB family, causing activation of the intracellular tyrosine kinase domain and receptor transphosphorylation. The newly formed phosphotyrosine residues act as docking sites for various adaptor molecules that consequently activate a number of intracellular signaling cascades, that, in case of constitutive activation of the pathway, leads to cell proliferation, inhibition of apoptosis, angiogenesis, and invasion/metastasis, resulting in tumor growth and progression [5].

Currently two main anti-EGFR strategies are in clinical use: low-molecular-weight TKIs that compete with ATP for binding to the tyrosine kinase portion of the receptor, and monoclonal antibodies that are directed at the ligand-binding extracellular domain thereby preventing ligand binding, receptor dimerization, and receptor signaling. These two classes of agents have shown solid preclinical and clinical activity in a variety of tumor types [6].

Among the receptor TKIs, single-agent erlotinib (Tarceva, Genentech, Inc, South San Francisco, CA, USA and OSI Pharmaceuticals Inc., Melville, NY, USA) improves survival in advanced NSCLC patients who progressed after chemotherapy [7-10] and is superior to chemotherapy in the first-line treatment of lung adenocarcinoma with an EGFR mutation in exon 19/21 [2]. The aggregated clinical experience today indicates that only patients whose tumors contain a sensitizing mutation in the EGFR tyrosine kinase domain derive an important and meaningful clinical benefit from these agents. Some randomized studies indicate that in patients not selected for such mutations these drugs might even have an adverse effect on outcome [11]. In an unselected patient population, gefitinib maintenance therapy also failed to show a survival advantage [11].

Not all patients with tyrosine kinase domain mutations respond to these inhibitors and even patients that respond usually only achieve a partial remission. In addition, some base-line mutations, for example those located in exon 20 of the kinase domain, are resistant or only weakly sensitive to current anti-EGFR TKIs. The efficacy of the inhibitors is also limited in time due to, in nearly half of the cases, the appearance of cells with a second "resistance" mutation, usually T790M located in the receptor tyrosine kinase domain [4]. An additional mechanism is the activation, either at baseline or acquired, of c-Met over-expression. Afatinib (BIBW 2992, Boehringer Ingelheim GmbH), an irreversible dual inhibitor of EGFR and HER2 kinases, retains some activity in tumors with T790M mutations although at doses that are a log higher than what is needed for cancers with only a sensitizing mutation [12]. Afatinib is currently being evaluated in phase III trials [13-17].

The chimerical IgG1 mAb cetuximab (ERBITUX, ImClone Systems Incorporated, New York, NY, USA and Bristol-Myers Squibb Company, Princeton, NJ, USA) is the most comprehensively studied anti-EGFR antibody. By blocking the ligand-receptor interaction, cetuximab down-regulates EGFR signaling, thereby inhibiting cell proliferation, apoptosis, and angiogenesis [6]. Cetuximab in combination with chemotherapy has been approved by the FDA for the treatment of metastatic colorectal cancer (CRC) and in combination with radiotherapy or a platinum derivative for the treatment of locally advanced head and neck cancer (HNC) [18,19]. Cetuximab has modest activity as a single agent as well as in combination with docetaxel in patients with advanced, chemotherapy-refractory NSCLC [20]. A multinational, multicentre, open-label, phase-III trial has shown that addition of cetuximab to platinum-based chemotherapy improved outcome for patients with advanced NSCLC [21]. However, the effect is small and no clear predictive biomarker has been identified.

The limitations of the clinical results obtained with single agent EGFR TKIs or cetuximab justify the investigation of additional therapeutic strategies, including enhanced targeting of the EGFR. RNA interference (RNAi), has been extensively explored in recent years in many targets. The ability of small interference RNA (siRNA) sequences to modulate gene expression has provided a powerful tool with which to study gene function and is being explored in clinical trials [22,23]. However, the combined use of RNAi and other types of EGFR targeting has not been explored.

In the current study we investigated whether the combination of EGFR inhibitory agents with EGFR-specific siRNA increases the therapeutic efficacy. To this end, we have examined the effects of either treatment alone versus the combination, in a set of lung cancer cell lines differing in their genomic status.

## Methods

### Cell lines and reagents

The human NSCLC cell lines H292 (CRL-1848™, mucoepidermoid pulmonary carcinoma) was kindly provided by Prof Dr Filip Lardon (Universiteit Antwerpen-CDE Geneeskunde-Oncologie). H358 (CRL-5807™, bronchoalveolar carcinoma), HCC827(CRL-2868™, adenocarcinoma), H1650(CRL-5883™, adenocarcinoma; bronchoalveolar carcinoma), and H1975(CRL-5908™, adenocarcinoma) were obtained from the American Type Culture Collection (ATCC, Netherlands). The cell line H292 was reported to be an EGFR and KRAS wild-type cell line by others [24,25]. We confirmed the wild-type status for both genes using real-time RT-qPCR and sequencing analysis (data not shown). H358 is EGFR wild-type and is mutated at codon 12 of KRAS [26], and in addition has a homozygous deletion of p53 [27]. H1650 and HCC827 have an in-frame deletion in the EGFR tyrosine kinase domain (EGFR tyrosine kinase domainΔE746-A750, exon 19). H1650 cells have also a deletion of the 3' part of exon 8 and the entire exon 9 of PTEN, which causes loss of the protein [28] and in addition express the insulin-like growth factor receptor (IGF1R) [29]. The cell line H1975 has a sensitizing L858R kinase domain mutation in exon 21, but also a second mutation (T790M, *in cis*, in the kinase domain) rendering them resistant to the reversible TKIs gefitinib and erlotinib [30]. Moreover, these cells express the Met receptor but without gene amplification [31]. Table 1 summarizes the relevant genomic status of the different cell lines. All five cell lines were cultured in the same RPMI 1640 medium (Invitrogen Corp., Gent, Belgium), supplemented with 10% heat-inactivated fetal bovine serum (Perbio Science NV, Erembodegem, Belgium), 2 mM L-glutamine and 1 mM sodium pyruvate at 37°C in a humidified incubator with 5% CO_2_. TKIs gefitinib (AstraZeneca, Cheshire, UK), erlotinib (AstraZeneca, Cheshire, UK), and the EGFR+HER2 specific afatinib (Boehringer Ingelheim, Ingelheim, Germany) stocks of 10 mM were prepared in dimethyl sulfoxide (DMSO) and stored at -80°C. The EGFR-specific monoclonal antibody cetuximab (2 mg/ml) was purchased from Merck KgaA, Darmstadt, Germany and stored at 4°C. The drugs were diluted in fresh RPMI 1640 with a final concentration of DMSO less than 0.1% in all experiments.

**Table 1:** Relevant genotypic changes in the NSCLC cell lines studied

Cell lines	EGFR status	KRAS status	PTEN
HCC827	Exon 19 deletion	Wild-type	+
H292	Wild-type	Wild-type	+
H358	Wild-type	Mutant	+
H1650	Exon 19 deletion	Wild-type	-
H1975	Exon 21 L858R + exon 20 T790M	Wild-type	+

### siRNA transfection

The EGFR specific siRNA designed by Invitrogen (5' GCAAAGUGUGUAACGGAAUAGGUAU 3') targets a sequence starting at nucleotide 1247 and lying at the junction of exon 8 and 9 (ref. SKU#12938-076, Invitrogen Merelbeke, Belgium) [32]. The glyceraldehyde-3-phosphate dehydrogenase (GAPDH) positive control siRNA (used in preliminary experiments to optimize the siRNA transfection efficiency, data not shown) was also from Invitrogen (ref. SKU#12935-140 Stealth RNAi GAPDH Positive Control, Invitrogen Merelbeke, Belgium). The negative control siRNA was from Eurogentec (OR-0030-neg05, Eurogentec S.A., Liege, Belgium) and consists of a proprietary siRNA sequence not corresponding to any eukaryotic gene. Transfection was by mixing siRNA with 1.5 μl Lipofectamine™2000 (Cat. No. 11668-019, Invitrogen Merelbeke, Belgium) for a final volume of 100 μl RPMI including 10% serum but without antibiotics. The procedure was according to the manufacturer. A positive control for transfection efficiency was the "TOX" transfection control, a proprietary RNA oligonucleotide that induces cell death, and siGLO Green, a modified, fluorescent RNA duplex that localizes to the nucleus (ref. D-001630-01-02 and D-001500-01-05, Thermo Scientific Dharmacon, Blenheim, UK).

### RT-qPCR

RNA isolation, RNA normalization, and reverse transcription were as described previously [32,33]. Intron-spanning RT-PCR primers (synthesized by Eurogentec S.A., Liege, Belgium) specific for EGFR or GAPDH mRNA were based upon GenBank sequence (EGFR, GeneID: 1956, NM_005228.3, and ENSEMBL sequence: ENST00000275493; GAPDH, GeneID: 2597, NM_002046.3, and Ensembl sequence: ENSG00000111640). They were designed using the Roche LightCycler Probe Design Software v1.0 followed by BLAST analysis http://blast.ncbi.nlm.nih.gov/Blast.cgi. The primer sequences were EGFR FWD: CGAGGGCAAATACAGCTT, EGFR REV: AAATTCACCAATACCTATT, and GAPDH FWD: TGAACGGGAAGCTCACTGG, GAPDH REV TCCACCACCCTGTTGCTGTA. The reverse primers were also used in the reverse transcription step. Real-time qPCR was performed in the Roche LightCycler^® ^1.5 instrument with SYBR green detection and melting curve analysis, as described previously [33]. The target mRNA abundance in each sample was normalized to its reference level as ΔCq = Cq_EGFR_-Cq_GAPDH_, where the Cq value is the quantification cycle number. The value ΔΔCq is the difference with a mock tranfected control. Experiments were performed in triplicate. The reduction of the EGFR mRNA level was expressed as a percentage and calculated with the formula: (1-1/2^ΔΔCq^) × 100 [32,34].

### Western blot analysis

After being treated with siRNA for the indicated periods, the cells were washed with PBS and lysed in a buffer containing 80 mM Tris-HCl (ph 6.8), 5% SDS, 10% glycerol, 5 mM EDTA (ph 8), 5% 2-MercaptoEthanol, 0.2% Bromophenolblue, and 1 mM phenylmethylsulfonyl fluoride. The lysates were centrifuged at 12,000 × *g *for 10 min at 4°C and boiled for 5 min. Twenty-five microgram protein of each sample was subjected to SDS-PAGE (8% SDS-acrylamide gel) and the separated proteins were transferred to hybond ECL nitrocellulose membranes (GE Healthcare Bio-sciences/Amersham, Diegem, Belgium) for 2 h at 100 mA. The membrane was incubated with a non-phospho-Tyr1173 EGFR antibody (1:1,000 dilution, clone 20G3, mouse monoclonal IgG_1К _, Bio-connect, Huissen, The Netherlands.) or a β-actin antibody (ref. A1978 AC-15 1:2,000 dilution, Sigma-Aldrich N.V. Bornem, Belgium). Primary antibodies were detected with an HRP-conjugated secondary antibody (1:4,000 dilution, ECL Anti-mouse IgG Peroxidse linked Na 931, GE Healthcare Bio-sciences/Amersham, Diegem, Belgium) and finally the membranes were subjected to chemiluminescence detection assay (ECL Plus Western Blotting Detection Reagents, GE Healthcare Bio-sciences/Amersham, Diegem, Belgium). Experiments were repeated in triplicate.

### Cell growth

Cell growth was assessed using a colorimetric tetrazolium (MTS) assay (CellTiter96 AQueous One Solution Cell Proliferation Assay G3580, Promega, Madison, WI, USA). The protocol was as follows: siRNAs, gefitinib, erlotinib, afatinib, or cetuximab were added to 96 well plates at increasing concentrations and incubated at 37°C for up to 72 h (96 h for siRNA) for single treatments. For the siRNA/TKI/antibody combinations, the agents were added to the cells first, and 24 h later the cells were transfected with EGFR siRNA in the same wells and incubated for another 48 h, because siRNA transfection efficiency is influenced by the agents if performed at the same time. Following addition of 20 μl of MTS reagent to each well, the plates were incubated for 2 h at 37°C in a humidified 5% CO_2 _atmosphere, and the absorbance at 490 nm was recorded using a 96-well microplate reader (Scientific Multiskan MK3, Thermo Finland). All assays were performed in triplicate. The results were the mean of six wells and expressed as the ratio of the absorbance of siRNA and/or agent treated wells/absorbance of mock control ×100.

### Cell viability

To further confirm the data from the above MTS assay, cell viability was detected by fluorimetric detection of resorufin (CellTiter-Blue Cell Viability Assay, G8080, Promega, Madison, WI, USA). The procedure was according to the manufacturer. The treatments and controls were as mentioned above. Fluorimetry (ex: 560 nm/em: 590 nm) was using an FL600 fluorescence plate reader (Bio-Tek, Winooski, Vermont, USA). All assays were performed in triplicate and each time six individual wells were used. Fluorescence data are expressed as the fluorescence of treated sample/mock control ×100.

### Caspase-3/7 activity detection

Caspase-3/7 activity was measured using a synthetic rhodamine labeled caspase-3/7 substrate (Apo-ONE^® ^Homogeneous Caspase-3/7 Assay, G7790, Promega, Madison, WI, USA) performed immediately after the detection of cell viability (described above) on the same wells, according to the instructions of the manufacturer. After incubation at room temperature for 60 min, the fluorescence of each well was measured (ex: 499 nm/em: 512 nm), using a FL600 fluorescence plate reader (Bio-Tek, Winooski, Vermont, USA). Caspase-3/7 activity is expressed as fluorescence of treated sample/mock control×100.

### Fluorescent microscopy evaluation of cell apoptosis and morphology

The effects of EGFR siRNA and different agents on apoptosis and nuclear morphology in the cells were assessed by Hoechst 33342 (Sigma-Aldrich N.V. Bornem, Belgium) and propidium iodide (PI, Sigma-Aldrich N.V. Bornem, Belgium) double fluorescent chromatin staining. In brief, after single or dual treatment of siRNA and/or agents, cells were washed with ice-cold PBS and stained 15 min with Hoechst 33342 (1 mg/ml) and PI (1 mg/ml), and observed under an advanced fluorescence microscope (ZEISS Axiovert 25, Zaventem, Belgium). Apoptosis and nuclear morphology were identified by condensation of nuclear chromatin and its fragmentation. This system determines the absolute number of viable cells (Hoechst 33342 positive/PI negative), early apoptotic cells (Hoechst 33342 positive/PI negative with blue fragmentations in the cells), late apoptotic cells (Hoechst 33342 positive/PI positive, with red fragmentations in the cells), necrotic cells (PI positive), and debris signals. Viable, apoptotic, and necrotic cells were counted in 10 different fields under the 200 × vision in each well in three independent experiments by two persons and the average result was compared to the mock control. Apoptotic cell numbers from different treatments were compared by being normalized to their viable cell numbers.

### Statistical analysis

SPSS19.0 was used for statistical analysis. Results were representative of three independent experiments unless stated otherwise. Values were presented as the mean ± standard deviation (SD). One-way Analysis of Variance (ANOVA) test was used to analyze significance between groups. The least significant difference (LSD) method of multiple comparisons with parental and control group was applied when the probability for ANOVA was statistically significant. Statistical significance was determined at a *P *< 0.05 level. In the analysis of additivity and synergism, the theoretical zero-interaction (exactly additive) dose-response curve for each siRNA + drug combination was calculated by applying the Bliss independence criterion [35,36]. Determination of possible synergy was also assessed by the Biosoft CalcuSyn program (Ferguson, MO, USA). The combination index (CI) was used to express synergism (CI < 1), additive effect (CI = 1), or antagonism (CI > 1) [37].

## Results

### Effects of EGFR-specific siRNA on target expression and malignant phenotype

**Figure 1:**
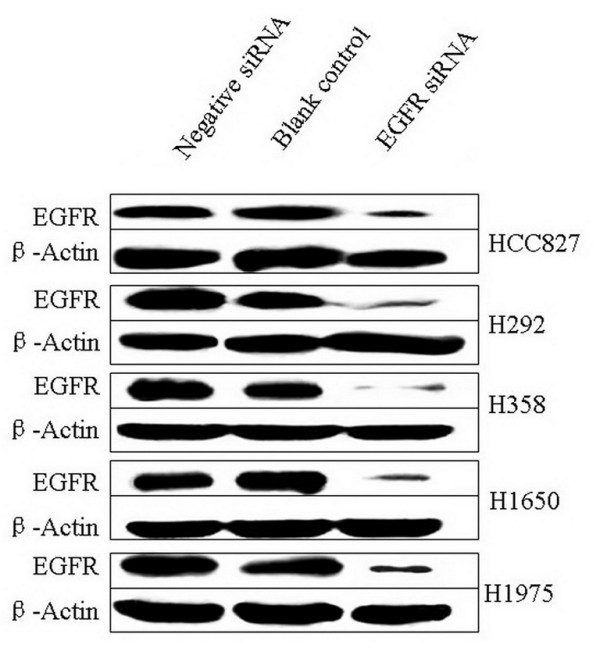
**siRNA induced EGFR protein down-regulation**. Down-regulation of EGFR protein was detected by western blot 72 h post transfection of 200 nM siRNAs.

Among different EGFR-specific siRNAs that were assessed for their ability to reduce EGFR mRNA levels, an efficient 25 bp "validated stealth" oligonucleotide from Invitrogen was chosen for its potent EGFR mRNA knock-down efficiency [32,33]. Transcript levels were detected by real-time RT-qPCR assay and relative quantification using GAPDH gene transcript as a reference. The knock-down ratios for the HCC827, H292, H358, H1650, and H1975 cell lines were in the same range: 83%, 87%, 82%, 88%, and 94%, respectively. The expression level of the EGFR protein was verified by immunoblotting, 72 h post transfection (Figure 1). EGFR expression in the cell lines transfected with EGFR-specific siRNAs was severely reduced compared to the negative control siRNA that had no effect. The EGFR-specific siRNA thus significantly inhibits EGFR mRNA and protein expression and with the same order of magnitude in all cell lines studied, independent of the genomic status of the EGFR.

**Figure 2:**
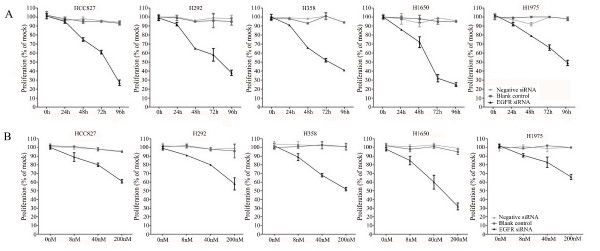
**Effect of EGFR siRNA transfection on the growth of the lung cancer cells**. Both time-dependent and dose-dependent effects were investigated. Cells were incubated in the presence of 200 nM siRNA for 0, 24, 48, 72, and 96 h **(A) **and also assessed with 0, 8, 40, or 200 nM siRNA for 72 h (dose-response curves, **B**) using the MTS cell growth assay. Cell growth is expressed relative to the mock control group as absorbance of siRNA/mock control ×100. The average ratio was calculated from six independent wells.

A colorimetric MTS tetrazolium assay (CellTiter 96, Promega, Madison, WI, USA) revealed that there was a time-dependent reduction of 50% or more of cell growth by the EGFR siRNA in all five cell lines. This was achieved within a 72-h time frame, except for the H1975 cell line carrying the T790M mutation that needed 96 h to achieve the same degree of inhibition. The steepest time response curve was in the H1650 cell line carrying both an exon 19 activating mutation and a PTEN mutation, and to a somewhat lesser degree in the H358 cell line carrying a KRAS mutation. Within a time frame of 72 h, a dose-dependent inhibition of cell growth was observed in all cell lines (Figure 2B). Again, the H1650 cells were the most sensitive and H1975 cells were the least sensitive cells (Figure 2A). To confirm the results assessed by the MTS assay, the effect on viability was assessed using a fluorimetric resorufin viability assay (CellTiter Blue, Promega, Madison, WI, USA), and by microscopic counting of viable (Hoechst 33342 positive/propidium iodide negative) cells. The results of both assays largely mirrored the MTS tetrazolium assay results (data not shown).

**Figure 3:**
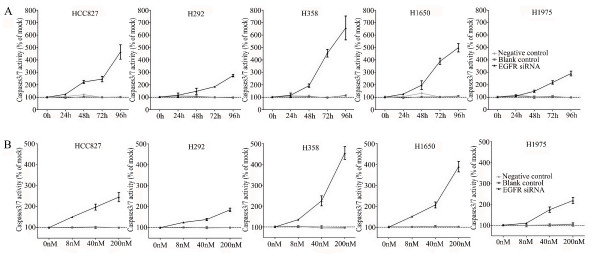
**Caspase 3/7 activity of the lung cancer cells with EGFR siRNA transfection**. Effects of siRNA treatment on caspase-3/7 activity. Cells were treated as mentioned in Figure 2, and the effect on caspase-3/7 activity was assayed and compared to the mock control in triplicate at 0, 24, 48, 72, and 96 h post transfection **(A)**. To accommodate the different cell numbers in the different treatments, caspase-3/7 induced fluorescence was normalized to resorufin fluorescence (viability) from the same sample.

To verify whether the EGFR siRNA is able to induce apoptosis, the CellTiter Blue assay was multiplexed with a fluorescent caspase 3/7 assay. The results show a time-dependent and dose-dependent caspase 3/7 signal in all cell lines (Figure 3). The most sensitive cell lines were the cell lines containing an exon 19 deletion (HCC827 and H1650) and the H358 cell line containing a KRAS mutation, while the H1975 and H292 cell lines required a significantly longer exposure and higher siRNA dose. In the H292 cell line even the highest concentration tested could not double the base line apoptotic level. A remarkable and unexpected high rate of apoptosis induction was observed in the cell line H358. The effect on apoptosis was confirmed microscopically by Hoechst 33342 and PI double fluorescent staining (data not shown). Again and surprisingly, in both assays the highest apoptotic signals were recorded for the H358 cell line, which is wild type for EGFR and carries a KRAS mutation that activates signaling downstream of EGFR (ERK/MAPK).

### Targeting EGFR with kinase inhibitors alone

**Figure 4:**
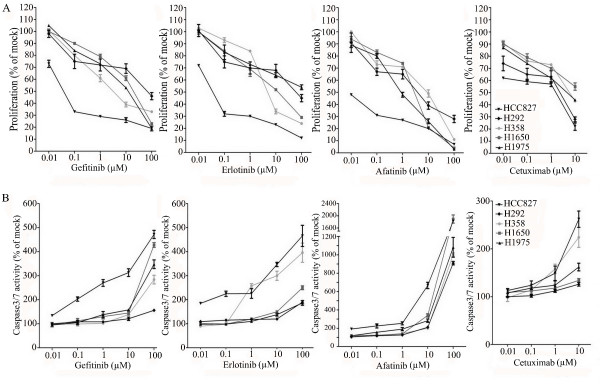
**Effect of TKI treatment on cell growth inhibition and apoptosis induction**. Effect of TKIs gefitinib, erlotinib, and afatinib and cetuximab on the growth **(A) **and caspase3/7 activity **(B) **of HCC827, H292, H358, H1650, and H1975 cells after 72-h incubation. Independent experiments were repeated six times and one representative result is shown. Points, mean value of six identical wells of a single representative experiments; bars, SD (n = 6).

All the cells were treated with reversible EGFR TKIs gefitinib and erlotinib, and the covalent inhibitor afatinib (targeting both EGFR and HER2), and with the monoclonal EGFR antibody cetuximab. The effects were studied in the colorimetric MTS tetrazolium proliferation assay (Figure 4A). By far the most sensitive cell line was HCC827, containing the exon 19 sensitizing mutation, with IC50 values ≤ 0.1 nM for the three kinase inhibitors. This was the case for the inhibition of cell growth as well as the induction of apoptosis (Figures 4A, B). The other cell lines lumped together and were 100- to 1,000-fold less sensitive to all three drugs, although subtle differences in sensitivity were observed. Among the three kinase inhibitors, afatinib had by far the highest molar potency in the sensitive HCC827 cell line, which was especially striking for the induction of apoptosis. With afatinib, a doubling of the apoptotic rate was already observed at the lowest concentration tested (10 nM). It is noteworthy that in H1975 cells carrying the T790M resistance mutation, afatinib had a slightly higher activity than the reversible kinase inhibitors, but this difference was small and the activity was still logarithmically inferior to what was observed in the HCC827 cell lines. With cetuximab an effect could be observed in all cell lines only in the supramicromolar concentration range, which is higher than the serum concentrations that are achieved at clinical dose levels, and thus these cell lines are all considered to be relatively resistant [38]. The effect of the TKIs and cetuximab was also studied using the fluorimetric resorufin viability assay, yielding analogous results (data not shown). Surprisingly, at relatively high concentration, starting from one micro molar concentration and up, erlotinib was able to induce caspase 3/7 signals in H358 cells (KRAS mutant, p53 minus) as high as in HCC827 cells.

### The effect of adding an EGFR specific siRNA to either EGFR TKIs or to cetuximab

**Figure 5:**
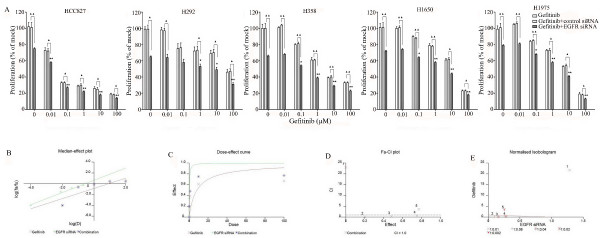
**Effect of adding an EGFR siRNA to gefitinib on cell growth inhibition**. Lung cancer cells were treated with gefitinib 24 h before the EGFR siRNA transfection. After transfecting the siRNA, the cells were incubated for another 48 h, after which cellular growth was assessed by the MTS assay. **(A) **shows the combination effect of gefitinib and siRNA. *P < 0.05 and **P < 0.01 compared to controls with only siRNA transfection. "black triangle" *P *< 0.05 and "double black triangles" *P *< 0.01 compared to agents plus control siRNA. Median-effect plot **(B)**, dose-effect curve **(C)**, Fa-CI plot **(D)**, and normalized isobologram (E) were computationally generated by Biosoft CalcuSyn program with EGFR siRNA and combined to gefitinib in H358 cells. Similar additive effect was found in all the combinations in all the five cell lines (data not shown).

**Figure 6:**
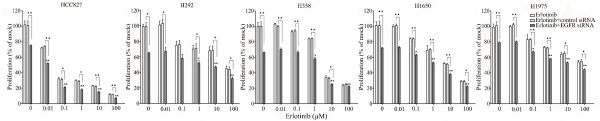
**Effect of adding an EGFR siRNA to erlotinib on cell growth inhibition**. Lung cancer cells were treated with erlotinib as mentioned in Figure 5. *P < 0.05 and **P < 0.01 compared to controls with only siRNA transfection. "black triangle" *P *< 0.05 and "double black triangles" *P *< 0.01 compared to agents plus control siRNA.

**Figure 7:**
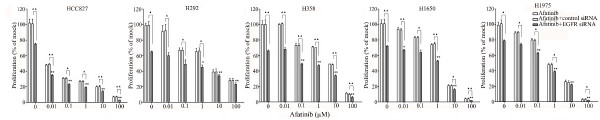
**Effect of adding an EGFR siRNA to afatinib on cell growth inhibition**. Lung cancer cells were treated with afatinib as mentioned in Figure 5. *P < 0.05 and **P < 0.01 compared to controls with only siRNA transfection. "black triangle" *P *< 0.05 and "double black triangles" *P *< 0.01 compared to agents plus control siRNA.

**Figure 8:**
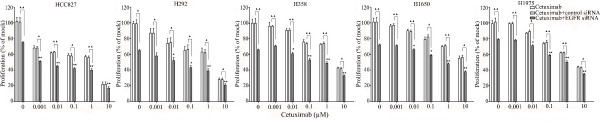
**Effect of adding an EGFR siRNA to cetuximab on cell growth inhibition**. Lung cancer cells were treated with cetuximab as mentioned in Figure 5. *P < 0.05 and **P < 0.01 compared to controls with only siRNA transfection. "black triangle" *P *< 0.05 and "double black triangles" *P *< 0.01 compared to agents plus control siRNA.

**Figure 9:**
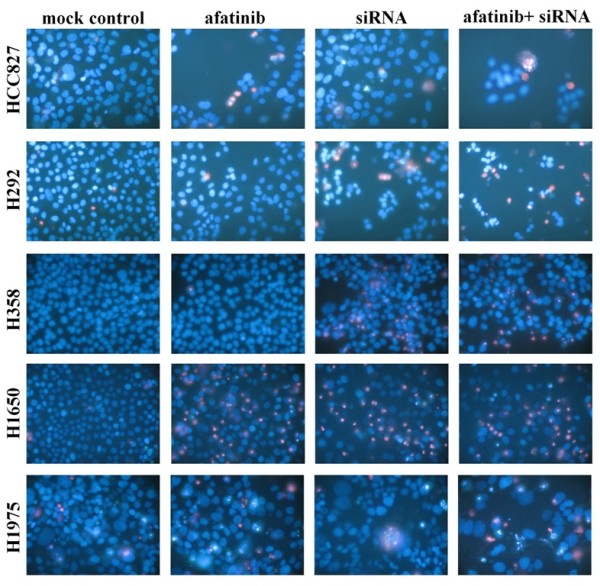
**Effect of combination of EGFR siRNA and afatinib with Hoechst 33342 and PI double fluorescent staining**. Effect of the combination of an EGFR siRNA and afatinib detected by Hoechst 33342 and PI double fluorescent staining. The concentrations were afatinib: 0.01 nM (assayed 72 h post treatment) and EGFR siRNA: 200 nM (assayed 48 h post transfection). Similar results were found with other concentrations of afatinib, and other drugs in all the five cell lines (data not shown).

**Figure 10:**
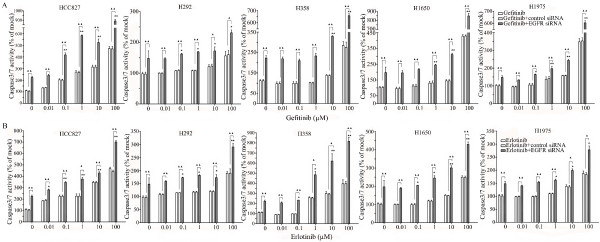
**Enhancement of apoptosis induction by the combination of EGFR siRNA plus gefitinib or erlotinib**. Lung cancer cells were treated with different drugs 24 h before the EGFR siRNA transfection. Forty-eight hours post transfection, the cell cultures were assayed for caspase-3/7 activity. (**A**: gefitinib; **B**: erlotinib). *P < 0.05 and **P < 0.01 compared to controls with siRNA transfection alone. "black triangle" *P *< 0.05 and "double black triangles" *P *< 0.01 compared to agents plus control siRNA.

**Figure 11:**
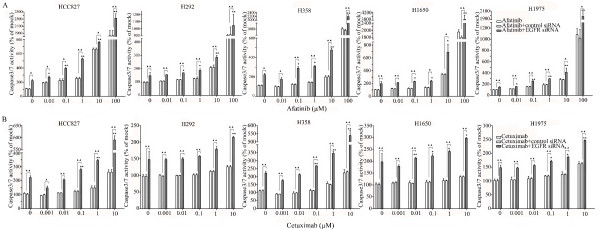
**Enhancement of apoptosis induction by the combination of EGFR siRNA plus afatinib or cetuximab**. Experiments were performed as mentioned in Figure 10. (**A**: afatinib; **B**: cetuximab). *P < 0.05 and **P < 0.01 compared to controls with siRNA transfection alone. "black triangle" *P *< 0.05 and "double black triangles" *P *< 0.01 compared to agents plus control siRNA.

The combination of siRNA with TKIs or cetuximab on cell growth was also studied using the colorimetric MTS formazan proliferation assay. The cells were first incubated with the TKIs or cetuximab. To avoid interference of these compounds with siRNA transfection, the transfection was carried out 24 h later. There was an enhancement of cell growth inhibition in all the five cell lines treated with the siRNA - drug combinations compared to either as a single agent alone. The most potent combination was the EGFR-specific siRNA plus afatinib (Figures 5, 6, 7, 8 and 9). As is observed in Figure 7, addition of siRNA with the concentration of 200 nM systematically further reduced cell growth in all cells over afatinib alone. Likewise, by comparing also zero afatinib dose with the samples treated with afatinib in increasing doses it is also apparent that the addition of afatinib to siRNA also increases the effect on growth. To ascertain the additive or synergistic nature, a combination index was calculated [35,36]. The results unambiguously show that the combined inhibition of proliferation is additive, since the combination indexes are close to or equal to one (Figures 5B, C, D, E). The additive effect was the weakest in the cell line HCC827, which is already the most sensitive to TKIs. This cell line is 10-fold more sensitive for growth inhibition to the combined action than the H292 and H358 cells and 100-fold more than the H1650 and H1975 cells. There was also a potentiation of apoptosis in all the five cell lines treated with the siRNA - drug combinations versus either as a single agent alone (Figures 10 and 11). The combined effect however is only clearly observed at doses between 10 and 100 nM of afatinib in cell line HCC827 and at supra micro molar doses of afatinib in the other cell lines. Again, the effect of the combinations of the drugs with siRNA was additive.

## Discussion

The use of EGFR TKIs is a clinically validated therapeutic option in NSCLC, especially for those tumors that harbor a sensitizing EGFR kinase domain mutation. However, single agent TKI treatment does not completely abrogate the oncogenic action of the receptor on cell growth and apoptosis induction. Furthermore, initial responders with mutant EGFR invariably develop secondary resistance to first generation TKIs [4,39,40]. Several strategies are being investigated for improving this therapeutic efficacy, by either combining EGFR TKI with other agents aimed at inhibiting other growth factor pathways that are responsible for EGFR TKI resistance, such as over-expressed c-Met. Another strategy is to target the EGFR with other agents that can suppress the oncogenic function, independent of the type of mutation. An example is cetuximab. Recently, the addition of cetuximab to afatinib has yielded impressive results in the treatment of EGFR reversible TKI resistant lung cancer due to T790M mutation [41].

EGFR-specific siRNAs may be good candidates for cancer therapy because of their specificity, efficiency, and endurance in gene-specific silencing and ability to suppress EGFR expression independent of the mutation status of the gene. Currently, there are only a few reports on the biological effects of EGFR siRNAs on lung cancer cells [31,42-45]. Sordella *et al. *[44] used a commercial EGFR wild-type siRNA pool that effectively induced the apoptotic enzyme caspase 3 at 96 h post transfection. The siRNA treatment also suppressed viability in H1975 cells expressing a T790M mutant EGFR and H1650 cells harboring a downstream PTEN mutation, but not in H358 cells that are wild-type for EGFR. In the present study, we have shown that an EGFR specific siRNA is very effective at suppressing the expression of EGFR in all cell lines tested, independent of the EGFR mutation status. We have also shown that all cell lines were variably inhibited in their growth by the siRNA and that the siRNA induced apoptosis in a dose- and time-dependent manner, upon transfection with siRNAs targeting wild type EGFR. Our results are partly in discordance with the data of Sordella *et al. *who, albeit using different siRNA sequences (commercially from Dharmacon, the concentration of siRNA was not reported) and detecting assays, found no biological effects in wild-type cells. These differences might reside in the respective concentration of the siRNAs used and the ability of the siRNAs to suppress gene expression which was high and uniform across cell lines in our experiments. Our results are in line with the report of Rothenberg *et al. *[43], which showed that lentivirus-based shRNA constructs targeting wild-type EGFR mRNA could promote cell death. Furthermore, a reduction in cell viability was observed in EGFR wild-type cells by Yamanaka *et al. *[45] who studied the effect of an EGFR siRNA (5'-GUCAGCCUGAACAUAACAU-3'), in different set of lung adenocarcinoma cell lines harboring a spectrum of EGFR wild-type, mutant, and KRAS mutant cell lines (PC-9 [EGFR 15-bp exon 19 deletion]; H3255 [EGFR L858R]; HT-1 [wt EGFR and wt KRAS]; 11-18 [wt EGFR and wt KRAS]; LK87 [KRAS G12D]; A549 [KRAS G12S]).

Although all cell lines tested in the present study were sensitive to our EGFR siRNA, some differences were noted. First of all, the differential sensitivity towards inhibition of cell growth versus apoptosis induction was not the same. The effect of an siRNA upon important aspects of the malignant phenotype, cell growth, and survival is a measure of the specific amplitude of the oncogenic potency and quality of the different mutations. The H1650 and HCC827 cell lines with an exon 19 deletion were the most sensitive, both for growth inhibition and apoptosis induction, confirming that the exon 19 mutation is the most oncogenic and addictive. H1650 cells have been described as resistant to TKIs due the loss of a functional PTEN suppressor [28]. Our results indicate that the EGFR mutation in H1650 cells at least partially bypasses the PTEN deficiency in driving cell growth and survival and that such a downstream mutation does not confer an absolute resistance to EGFR inhibition. To the contrary, upon siRNA treatment, this cell line was the second most sensitive to both growth and apoptosis induction. The lesser sensitivity of H1975 cells to EGFR siRNA treatment despite an equally high inhibition of EGFR protein expression indicates that the EGFR carrying a T790M mutation in combination with an exon 21 mutation is a less potent driver of cell growth and survival, which could also help to explain the clinical resistance to TKI inhibition of that receptor. Our siRNA results also confirm that in EGFR wild-type cells the receptor contributes the least to the malignant phenotype if at all, especially for cell survival. While there were anti-proliferative effects in the H292 cell line with a wild-type status, this cell line was relatively resistant to apoptosis induction. This is in concordance with the clinical experience that such cancers do not really benefit from TKI treatment. The most puzzling of our results are in the H358 cell line that has a wild-type EGFR receptor and carries a homozygous KRAS mutation that normally subverts the signaling emanating from the EGFR and creates resistance to inhibition of the receptor with TKIs or monoclonal antibodies [46,47]. In our experiments this cell line was the most sensitive to apoptosis induction and growth inhibition by siRNA EGFR inhibition. This result could not be explained by a higher EGFR mRNA knockdown in this cell line. H358 cells were found to be "KRAS-addicted" cells in which ablation of KRAS expression by shRNA interference results in apoptosis induction [48]. Inhibition of growth by EGFR siRNA has also been observed in KRAS mutant cell lines A549 and LK87 [45]. Our hypothesis is that the strong reduction of EGFR induced by EGFR-specific RNA interference, also induces a large depletion of GRB2-SOS complexes necessary to load GTP into normal or mutant KRAS and hence interferes with KRAS signaling. However, there are other, non-mutually exclusive possibilities. H358 cells were found to secrete increased levels of the EGFR ligand amphiregulin [49]. Knocking down EGFR expression would interrupt the amphiregulin/EGFR positive feedback loop and this could induce apoptosis. Thirdly, H358 cells were found to have a high ErbB3 expression [50], and since EGFR links to PI3K signaling via ErbB3, the PI3/AKT pathway may also be a major source of malignant growth in these cells (in addition to KRAS). The removal of PI3K/AKT signals by EGFR RNAi might then also lead to apoptosis. Moreover, others have reported observations that might point in the same direction as the present study: Sunaga *et al.*[49] found that cell survival is not much affected by KRAS knockdown in KRAS mutant NSCLC cell lines and hypothesized that a feedback signal to EGFR and Akt leads to increased stimulation. An additional mechanism for the observed effect might be an off-target effect of erlotinib on the Janus kinase 2 (JAK2). Erlotinib was shown to decrease phosphorylation of JAK2 and STAT-5 in EGFR-negative myelodysplastic syndrome (MDS) cell lines KG-1 [50] and erlotinib can disrupt signaling of the JAK2/STAT-5 pathway. JAK2 is activated by mutant p53 (or p53 deficiency) [51]. Thus, some of the survival pathways emanating from EGFR bypass KRAS in the cell line H358, and the KRAS mutation is more important for resistance to proliferation and less for apoptosis induction. Our and others' results suggest that the presence of a KRAS mutation (or other unknown alterations in these cells) could render H358 cells dependent on EGFR signaling and that EGFR would be a candidate therapeutic target in such cancers. In the current work we have explored the effects of a near maximal elimination of EGFR using siRNA. Although our experiments do provide an estimate of the relative oncogenic potency of the various EGFR mutations and downstream mutations, currently we do not know whether it will be possible to attain similar concentrations of a therapeutic equivalent of our siRNA *in vivo *and in patients and thus obtain similar efficacy.

It is within that window of a maximal effect of EGFR inhibition that we have to analyze the results with TKI or cetuximab inhibition, which are strikingly different. The effect of TKI inhibition on the malignant phenotype is indeed the integration of several variables: the oncogenic potency of the targeted receptor, the significance of the kinase activity to this oncogenic potency, the variable sensitivity of the receptor to kinase inhibitors and the relative potency of kinase inhibitors to shut down this enzymatic activity. The action of monoclonal antibodies is even more complex and more difficult to relate to the mutational status of the receptor. By analogy to what is observed in the clinical studies, the exon 19 deletion HCC827 cell line conferred by far the highest sensitivity to TKI which is consistent with earlier reports [4,29,39,42,52-55]. This is also consistent with the high dependency of this cell line on this mutant receptor for cell growth and survival in our siRNA experiments. Comparatively, all other cell lines are to be considered to be relatively resistant to TKI inhibition. The striking difference with the siRNA results for the two cell lines with downstream TKI resistance mutations (PTEN and KRAS) indicates that the kinase activity of the receptor is not the sole mediator of the oncogenic activity of EGFR, although we observed some reflection of the siRNA results in the KRAS mutant H358 cells, especially with higher concentrations of erlotinib with regard to apoptosis induction. None of the cell lines had a relevant sensitivity to cetuximab alone under 10% FBS culture condition, and even the TKI sensitive cell line HCC827 cells showed limited response. This might be explained by the absence of an oncogenic significance of the wild-type receptor and insensitivity of mutant receptors to inhibition by monoclonal antibodies. Activating mutations indeed confer hypersensitivity to TKIs, but not necessarily to inhibition by monoclonal antibodies [56]. The failure to detect a significant activity for cetuximab agrees with the absence of a significant activity as single agent or very modest added benefit in clinical lung cancer in association with chemotherapy [21].

Although EGFR is clearly a valid target in NSCLC treatment, the efficacy demonstrated by EGFR-targeted agents is not maximal as shown in preclinical models and more recently in clinical trials [40,41]. One approach to improve responsiveness to EGFR inhibitors may be to simultaneously target multiple HER family members. Afatinib is currently the most advanced compound in this class. Afatinib is an irreversible EGFR/HER2 inhibitor, with activity against wild-type and mutant forms of EGFR [57]. Afatinib was more potent than gefitinib, erlotinib, and lapatinib in inducing the cell death of NSCLC cell lines, including those harboring wild-type EGFR, and the erlotinib-resistant T790M mutation [57]. It was also found in the present study (Figure 4) that the molar potency of afatinib against these cells was significantly higher than either gefitinib or erlotinib. HCC827 cells harboring the activating E746_A750 deletion were highly sensitive to afatinib, whereas other NSCLC cell lines were moderately sensitive, which is in agreement with other reports [57,58]. The activity against the resistance mutation T790M and cell lines with downstream resistance mechanisms was, however, only slightly better than the reversible TKIs.

The several EGFR-targeting approaches differ in action mechanisms. TKIs compete with ATP to bind to the EGFR kinase, hence inhibiting EGFR autophosphorylation and activation of downstream signaling. Anti-EGFR antibodies prevent receptor dimerization and hence activation [59]. However, none of these agents alone does maximally suppress EGFR signaling or the effect of mutant EGFR in the malignant phenotype, as also shown in our experiments. The combination of cetuximab with the different TKI has already been tested [60,61]. The *in vitro *and *in vivo *results showed that the combined treatment can augment the potency of EGFR signaling inhibition. Ramalingam *et al. *[62] used a combination of cetuximab and gefitinib for patients with advanced/metastatic lung cancer who were previously treated with platinum-based chemotherapy. It was concluded that dual inhibition is feasible and safe, and may have modest activity in advanced/metastatic NSCLC. The combination of afatinib and cetuximab can even overcome resistance due to the T790M mutation both preclinically [40] as well as clinically [41]. In the present study, the combined treatment of EGFR siRNA and TKIs or antibody achieved increased tumor cell growth suppression (about 30% more at the most) (Figures 5, 6, 7, 8, and 9) in all the five NSCLC cell lines and increased apoptosis as high as by 100% (Figures 10 and 11). The effect with the different agents in the different cell lines was additive, not synergistic, as calculated by a combination index (CalcuSyn software). Again, the differential sensitivity of the cell lines to the combination mimicked their sensitivity to TKI alone: the cell lines that demonstrated the most sensitivity to siRNA had the largest effect from the combination, including the cell lines with downstream TKI-resistance mutations (which also in this context displayed the highest sensitivity) or the T790M mutation. The least added effect was seen with afatinib associated to EGFR siRNA in the cell line with the TKI sensitive exon 19 deletion mutation, in which afatinib alone is already highly active at very low molar concentrations.

## Conclusions

We conclude that RNA interference by siRNA oligonucleotides should be further explored and developed as a therapeutic modality in the treatment of EGFR mutant lung cancer and also including KRAS mutant lung cancer, and lung cancers containing the resistance mutations T790M or downstream pathway activation such as PTEN inactivation. As already mentioned, it is not known whether the concentration of siRNAs or an equivalent of this used in the present study will be achievable *in vivo *and in the clinic. EGFR siRNAs or a similar technology that eliminates the receptor protein physically from the cancer cell could help to improve the treatment results in difficult to treat lung cancers. Methods of *in vivo *siRNA delivery are currently being researched, and some reports have already described the systemic use of siRNA in cancer patients [63]. The most appealing small molecule to test in a combination strategy would be afatinib, the irreversible EGFR/HER2 inhibitor.

## Competing interests

The authors declare that they have no competing interests. The project was partly funded by a research grant from Boehringer Ingelheim.

## Authors' contributions

GC carried out the primer and siRNA designing, transfection of the siRNA, RNA extraction, real-time PCR and protein, proliferation, viability, apoptosis and western blot experiments, and drafted the manuscript. PK conceived of the study, carried out primer and siRNA designing, supervision of all the experiments, and helped to draft the manuscript. ET participated in the design of the study and interpretation of data, and helped to revise the manuscript. IAU participated in cell culture, drugs treatment, western blot experiments and contributed to the revising of the manuscript. JDG participated in the design of the study and coordination and helped to draft and revise the manuscript. All authors read and approved the final manuscript.

## Pre-publication history

The pre-publication history for this paper can be accessed here:

http://www.biomedcentral.com/1741-7015/10/28/prepub
