# Correction to: Targeting the epidermal growth factor receptor in non-small cell lung cancer cells: the effect of combining RNA interference with tyrosine kinase inhibitors or cetuximab

**DOI:** 10.1186/s12916-020-1501-8

**Published:** 2020-02-01

**Authors:** Gang Chen, Peter Kronenberger, Erik Teugels, Ijeoma Adaku Umelo, Jacques De Grève

**Affiliations:** 1 Laboratory of Medical and Molecular Oncology and Department of Medical Oncology, Universitair Ziekenhuis Brussel, Vrije Universiteit Brussel, Laarbeeklaan 101, 1090 Brussels, Belgium; 2 Department of Pathology, First Affiliated Hospital, Guangxi Medical University, Shuangyong Road 6, 530021 Nanning, Guangxi People’s Republic of China; 3 Department of Gezondheidszorg, Erasmushogeschool Brussel, Laboratory for Biotechnology, Laarbeeklaan 125, 1090 Brussels, Belgium

**Correction to: BMC Med (2012) 10:28**

**https://doi.org/10.1186/1741-7015-10-28**

Following publication of the original article [1], the authors reported that there was an error in Fig. 9, which contained a misplaced picture. The authors confirm that all of the published results and conclusions of the paper remain unchanged, as well as the figure legends. The authors apologize for any confusion caused. The corrected Fig. 9 is shown as follows:

**Fig. 9:**
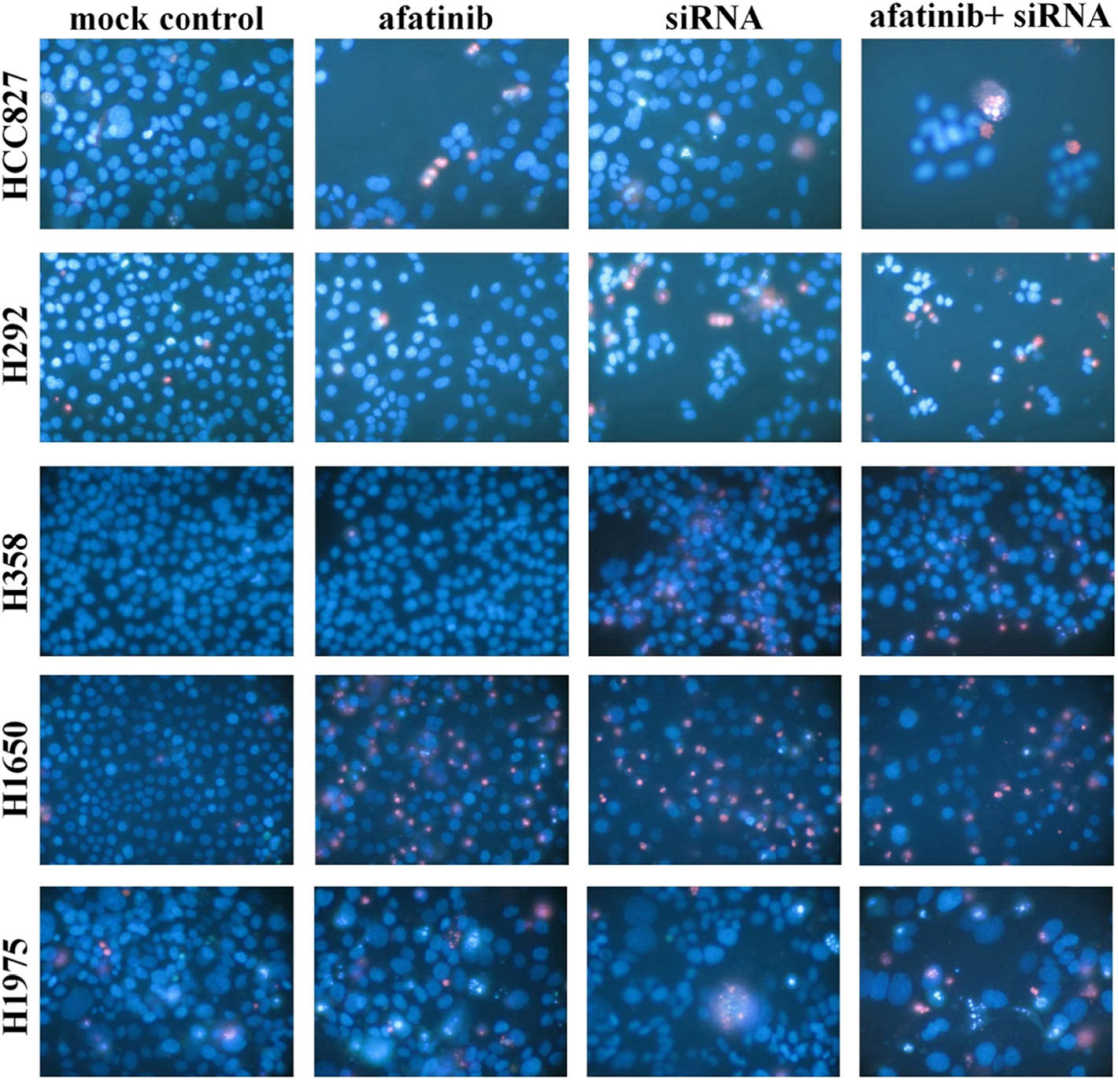
Effect of combination of EGFR siRNA and afatinib with Hoechst 33342 and PI double fluorescent staining. Effect of the combination of an EGFR siRNA and afatinib detected by Hoechst 33342 and PI double fluorescent staining. The concentrations were afatinib: 0.01 nM (assayed 72 h post treatment) and EGFR siRNA: 200 nM (assayed 48 h post transfection). Similar results were found with other concentrations of afatinib, and other drugs in all the five cell lines (data not shown).
